# Acquiring complex concepts through classification versus observation

**DOI:** 10.1186/s41235-024-00608-z

**Published:** 2024-12-16

**Authors:** Daniel Corral, Shana K. Carpenter

**Affiliations:** 1 Department of Psychology, Syracuse University, 346 Marley Education Building, Syracuse, NY 13244 USA; 2 Oregon State University, Corvallis, USA

**Keywords:** Classification learning, Observation learning, Complex concept acquisition, Explanation feedback, Retrieval practice, Transfer of learning, Learning and instruction

## Abstract

We report six experiments that examine how two essential components of a category-learning paradigm, training and feedback, can be manipulated to maximize learning and transfer of real-world, complex concepts. Some subjects learned through classification and were asked to classify hypothetical experiment scenarios as either true or non-true experiments; others learned through observation, wherein these same scenarios were presented along with the corresponding category label. Additionally, some subjects were presented correct-answer feedback (the category label), whereas others were presented explanation feedback (the correct answer and a detailed explanation). For classification training, this feedback was presented after each classification judgment; for observation training this feedback was presented simultaneously with the hypothetical experiment. After the learning phase, subjects completed a posttest that included one task that involved classifying novel hypothetical scenarios and another task comprising multiple-choice questions about novel scenarios, in which subjects had to specify the issue with the scenario or indicate how it could be fixed. The posttest either occurred immediately after the learning phase (Experiments 1–2), 10 min later (Experiments 3–4), two days later (Experiment 5), or one week later (Experiment 6). Explanation feedback generally led to better learning and transfer than correct-answer feedback. However, although subjects showed clear evidence of learning and transfer, posttest performance did not differ between classification and observation training. Critically, various learning theories and principles (e.g., retrieval practice, generation, active learning) predict a classification advantage. Our results thus call into question the extent to which such theories and principles extend to the transfer of learning.

**Significance statement**

When learning to recognize a given concept, two training approaches that are often used is to have people either learn by classifying examples of the concept (classification training) or by having them study such examples (observation training). However, it is unknown which of these approaches better aid learning. Generally, tasks that encourage active learning improve learning outcomes, whereas those that overly strain peoples’ mental capacity hinder learning outcomes. Classification training seems to engage various active learning strategies (e.g., retrieval practice, hypothesis testing), but might be cognitively effortful, whereas observation training might be more passive, but might place less cognitive strain on learners. We report six experiments that examine which of these two training methods lead to superior learning of ecologically valid, complex learning materials. Although subjects showed evidence of clear learning and transfer across these six experiments, this benefit was no different between classification and observation training. Critically, various learning theories and principles (e.g., retrieval practice, generation, active learning) predict a classification advantage. Our results thus call into question the extent to which such theories and principles extend to the transfer of learning. Our results also suggest that for the learning of complex concepts, both classification and observation training can be effective. 

## Introduction

On a daily basis, students must learn to recognize a multitude of concepts across novel scenarios. These scenarios can greatly differ superficially from the scenarios that were encountered when the corresponding concept was learned. For example, a student in a statistics course might learn about a *type one error*—rejecting the null hypothesis when it is true—occurring in a research context, but might then be required to recognize this concept in a medical or legal context (for examples, see Corral et al., 2019). Similarly, students in a research methods course might need to learn to recognize and distinguish scenarios that involve true from non-true experiments across a wide array of domains, which can differ quite considerably from one another (e.g., physics, psychology, biology, chemistry, mathematics, anthropology).

**Fig. 1:**
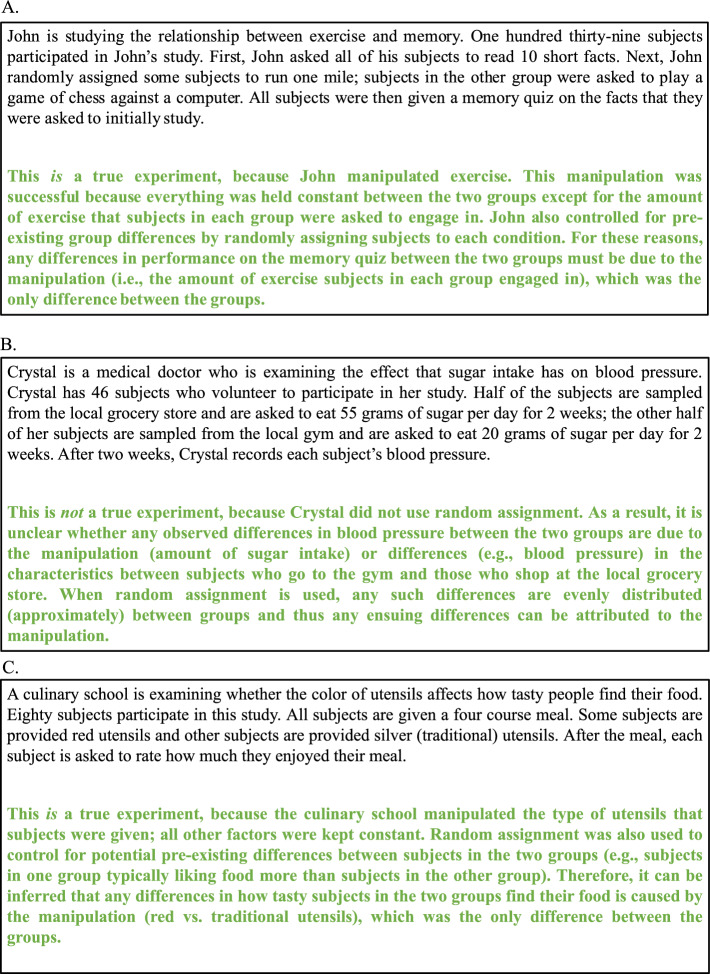
Hypothetical experiment scenarios (with explanation feedback) from the domains of psychology (Panel **A**; true experiment), medicine (Panel **B**; non-true experiment), and food tasting (Panel **C**; true experiment), which were used as stimuli in Experiments 1–6

Consider the three hypothetical experiment scenarios in Fig. 1 (taken from Corral & Carpenter, 2020; also see Corral et al., 2021), which correspond to three domains of research (psychology, medicine, and food management). A student might learn about the principles of true experiments (e.g., random assignment) from scenarios that share some surface similarities with the scenario in Fig. 1A (psychology) but might need to recognize these concepts and apply and transfer them to other domains, such as the scenarios shown in Fig. 1B (medicine) or 1C (food tasting).

## The problem of transfer

Considerable research has shown that superficial differences between example scenarios used during training and novel, analogous scenarios can greatly impair the transfer of learning (Barnett & Ceci, 2002; Detterman, 1993; Gick & Holyoak, 1983; Holyoak & Koh, 1987; Melby-Lervåg & Hulme, 2013; Reed, 1984, 1989; Reeves & Weisberg, 1994; Ross, 1987, 1989). This issue has been well documented in the problem-solving literature, wherein learners seemingly learn a problem type’s solution strategy, but are unable to apply it to novel, analogous problems (Corral et al., 2023; Mayer, 1998; Mayer et al., 1995). This phenomenon is known as the *inert knowledge problem* (Whitehead, 1929) and has been posited to be the primary impediment to the transfer of learning (Corral & Kurtz, 2024).

The inert knowledge problem seems to primarily arise because learners fail to recognize that a novel target scenario is analogous to previous example scenarios that have been encountered (Corral & Kurtz, 2024). Indeed, when learners are asked to think about how the target problem relates to previous (analogous) example problems, they are typically able to solve the target problem (Butler, 2010; Gick & Holyoak, 1983; Holyoak & Koh, 1987). These findings suggest that the corresponding solution strategy had indeed been learned, but that learners simply failed to realize that it was applicable to the problem at hand. One plausible way to improve the transfer of learning is thus to help learners better recognize when a previously learned concept is instantiated in a novel scenario (Corral & Kurtz, 2024).

One question to consider is how instructors can better teach students to accomplish this goal. To answer this question, it is first important to note that scenarios often contain many irrelevant properties, which can obscure the underlying concept(s) they represent (Anderson, 1993; Sweller et al., 1983). Recognizing that superficially different scenarios instantiate a common concept thus requires generalizing or abstracting knowledge about that concept, such that learners can look beyond the superfluous properties of a scenario and recognize that it contains the necessary elements that define the concept. For example, although the scenarios in Fig. 1A and C consist of many different surface features, they are true experiments because in both cases only the variables of interest were manipulated, and all other factors were held constant between or among the conditions. Recognizing that both scenarios instantiate the concept of a true experiment thus requires that learners can recognize that both scenarios meet the criteria of a true experiment, despite their superficial differences. This recognition process presumably underlies making a classification judgment, as recognizing that two scenarios instantiate the same concept is akin to indicating that they are members of the same category—a true experiment.

## Facilitating transfer through category learning

Theoreticians have posited that a typical category-learning paradigm is particularly well-suited for helping learners recognize to-be-learned concepts across superficially different scenarios (Corral & Kurtz, 2024). In a typical category-learning paradigm, on each trial learners are presented a stimulus and are asked to classify it. After responding, subjects are typically presented *corrective feedback*, wherein they are shown the correct answer along with whether their answer was correct. Learners are then shown a new stimulus on the next trial, which typically differs superficially from the previous stimuli; this process continues until some criterion is met (e.g., a specified number of trials is completed, a learning criterion is reached). Learners thus have the opportunity to view many examples that instantiate the to-be-learned concepts and receive extensive practice at recognizing and classifying such concepts across various superficially different scenarios.

Although this paradigm is relatively simple, it nevertheless consists of various components that leverage many well-established learning principles and strategies (see Corral & Kurtz, 2024), such as the use of varied examples (Butler et al., 2017), interleaving of examples from different to-be-learned categories (Eglington & Kang, 2017; Kang & Pashler, 2012; Kornell & Bjork, 2008), spaced study (Carpenter et al., 2022; Dempster, 1988; Gerbier et al., 2015; Glenberg, 1979; Vlach, 2014), retrieval practice (Carpenter, 2009, 2011, 2012; Carrier & Pashler, 1992; Dunlosky et al., 2013; Roediger, 2013), and feedback (Benassi et al., 2014).

Importantly however, it is an open question as to how these principles can best be incorporated into a category-learning paradigm. Although the literature on category learning spans many decades, this work has mostly used category learning as a tool to test theories on concept learning and representation and has not carefully investigated how the paradigm itself can be used and modified to improve learning and transfer. To this end, the present paper examines this question and focuses on two of the more prominent components of a traditional category-learning paradigm: (a) *classification* and (b) *feedback*.

### Classification

Classification incorporates four key learning strategies: (1) learners engage in hypothesis testing, as they can generate hypotheses about the elements that define the to-be-learned categories, which is posited to increase attention and engagement (Hunt, 1965; Johnson & Krems, 2001; Klahr & Dunbar, 1988; Markant, 2019; Markant & Gureckis, 2014), (2) the process of generating hypotheses incorporates principles of the *generation effect*, which should increase attention, engagement and motivation (Chechile & Soraci, 1999), (3) learners engage in extensive retrieval practice, which can enhance the learning and memory of the information that is retrieved (Butler, 2010; Carpenter, 2009, 2011, 2012; Carrier & Pashler, 1992; Dunlosky et al., 2013; Roediger, 2013), as learners can actively retrieve their hypotheses on each trial, along with category examples from previous trials, and (4) learners can compare the retrieved examples from previous trials to the example on the present trial, which incorporates the powerful learning benefits of comparison (Alfieri et al., 2013; Gentner & Medina, 1998; Gentner & Namy, 1999; Kotovsky & Gentner, 1996).

### Feedback

Feedback allows learners to continually check and update their category hypotheses based on whether their response was correct. Feedback is essential to learning and transfer (Benassi et al., 2014) and has been shown to be necessary for learning complex concepts (Corral & Carpenter, 2020; Corral et al., 2021).

Critically, there are various ways in which classification and feedback can be implemented in a category-learning paradigm. For example, learners can make classification judgments (classification training), or they can study examples with the corresponding category label (observation training). Similarly, there are various ways in which feedback can be presented to learners during category learning. Although learners are typically provided corrective feedback during category learning, they can also be presented *explanation feedback*, in which corrective feedback is coupled with an explanation of the correct answer (see Corral & Carpenter, 2020 and Corral et al., 2021).

Previous work has shown that explanation feedback leads to superior learning and transfer during category learning than corrective feedback (Corral & Carpenter, 2020 and Corral et al., 2021). What is less clear, however, is how these types of feedback might interact with classification versus observation training. Given the close relationship between the classification and feedback components in category learning, it is important to understand how the two might interact with one another. The present paper therefore examines two central questions: (a) which type of training, classification versus observation, leads to better learning and transfer during category learning? and (b) does this outcome vary as a function of whether learners receive correct-answer versus explanation feedback?

## Classification versus observation

As noted earlier, classification can engage retrieval (Corral & Kurtz, 2024). Thus, based on principles of retrieval practice (Carpenter, ; Carrier & Pashler, 1992; Dunlosky et al., 2013; Karpicke et al., 2014), learning and transfer should be best for learners who receive classification training compared to observation training. A similar prediction can be derived from extant work on category learning, wherein classification is posited to highlight diagnostic attributes of the to-be-learned categories, which should thus better aid subsequent classification (Markman & Ross, 2003; Yamuchi & Markman, 1998, 2000). In line with these predictions, some studies have shown benefits of classification training over training that involves observation (Ashby et al., 2002; Carvalho & Goldstone, 2015; Estes, 1994; Jacoby et al., 2010; Love, 2002; Markant & Gureckis, 2014; Ramscar et al., 2010; Steininger et al., 2022; Yang & Shanks, 2018).

On the other hand, it is possible that making a classification judgment is more cognitively demanding than observation training, as learners must engage in multiple reasoning tasks on each trial. First, prior to making a classification judgment, learners must select a hypothesis or a set of hypotheses to consider. Because the learner does not know which category the stimulus on that trial is a member of, they must consider hypotheses for each of the to-be-learned categories. Learners must then retrieve these hypotheses, hold them in working memory, and examine whether the stimulus on that trial accords with each of the hypotheses that are being considered. After making a classification judgment, the learner must then examine the feedback that they receive, consider whether their response was correct, which requires remembering their response, and then update their hypotheses accordingly. Learners’ attention is thus divided across all of these tasks during classification learning.

We also note that conceptually related work on problem solving has shown that when trying to learn the elements that define a given problem category, learners often focus on superfluous features, which can obscure the category’s underlying structure (Anderson, 1993; Sweller, 1988; Sweller et al., 1983; also see Chi et al., 1981). As a result, during the process of hypothesis testing during classification training, learners might develop the wrong hypotheses about what determines category membership. These learners might thus expend more cognitive resources testing a greater number of incorrect hypotheses and subsequently correcting said hypotheses than learners who receive observation training.

In instances when learners select the wrong category and their hypothesis is incorrect, after receiving feedback, they must figure out what aspect(s) of their hypothesis were incorrect. After doing so, learners must update said hypothesis accordingly and hold this update in memory. Complicating matters further is that incorrect hypotheses might persist and might be reinforced in cases where a classification response is incidentally correct, as the learner may erroneously think that their hypothesis led to the correct response. This issue can lead to proactive interference, even after the category has been learned (Corral & Jones, 2024) and might require greater effort and attention to override later in learning, which might further deplete cognitive resources.

In contrast, in observation training, the stimuli are presented with the corresponding category label. Learners can thus focus exclusively on hypotheses that correspond to the category that is specified on that trial. As a result, observation learners can consider a smaller set of hypotheses than learners who receive classification training (as classification learners do not know the category of the stimulus prior to making a classification judgment and might therefore have to consider hypotheses from all of the to-be-learned categories). Moreover, observation learners do not need to make a classification judgment, nor do they need to consider whether their response was correct, since no overt response is required of them. Observation learners therefore do not need to shift their attention between the correctness of their response and the elements in the stimulus.

Due to these differences between classification and observation training, the former might divide learners’ attention to a greater extent than the latter and consequently place a greater load on working memory. Based on cognitive load theory (Sweller, 1988; see also Carpenter et al., 2020), which holds that tasks that place a high load on working memory can impair learning, observation training might be expected to lead to better learning and transfer than classification training.^1^

It has also been posited that classification training leads to a narrow focus of attention, wherein learners largely focus on the discriminative elements of the to-be-learned categories and ignore non-diagnostic elements, and once these diagnostic elements are acquired, learners stop attending to other elements of the categories (Levering & Kurtz, 2015). In contrast, this work has proposed that observation training leads to a broader focus of attention, as learners focus on both discriminative and non-discriminative elements of the to-be-learned categories and thus continue to learn about such categories even once the discriminative elements have been acquired. Learners who receive observation training might therefore learn more detailed nuances about the to-be-learned categories than learners who receive classification training. In line with these ideas, some studies that have compared classification to observation training have actually found evidence of an observation advantage (Levering & Kurtz, 2015; Patterson & Kurtz, 2020).

### Type of feedback

A core question to consider is how the type of feedback that subjects receive during category learning impacts learning through classification and observation training. Although typical category-learning paradigms use corrective or correct-answer feedback, there are various other options. For instance, as previously noted, explanation feedback typically leads to better concept learning than both corrective (Corral & Carpenter, 2020) and correct-answer feedback (Corral et al., 2021).

Nevertheless, it is important to note that explanation feedback includes more information that learners must process than both corrective and correct-answer feedback. To explain further, when learners receive explanation feedback, they must also compare the hypotheses that they are considering and determine how those correspond with the feedback that they are presented. Thus, pairing classification training, which might place a greater load on learners than observation training (as explained earlier), with explanation feedback might be particularly taxing for learners. For this reason, combining classification training with explanation feedback might weaken the benefits of explanation feedback relative to explanation feedback paired with observation training.

Alternatively, it is possible that this pairing (i.e., classification training with explanation feedback) might be best for learning, as it might maximize engagement and attention. Classification training with explanation feedback requires (a) generating hypotheses, (b) testing them through classification judgments, (c) considering how the explanation feedback corresponds with said hypotheses, (d) updating said hypotheses, and (e) then testing these hypotheses once more. This process might better engage attention than other methods of category learning that are less involved (e.g., classification training with correct-answer feedback, observation learning with explanation feedback).

Another possibility is that pairing observation learning with explanation feedback is best for learning, as learners are shown a detailed explanation of the correct answer, which lessons their opportunity to develop incorrect hypotheses. As such, observation training with explanation feedback might lead to less proactive interference from incorrect hypotheses that are formed during training than other methods of category learning (e.g., classification training with correct-answer feedback). In addition to this benefit, observation training with explanation feedback seems to involve fewer subtasks than other approaches (e.g., classification training with explanation feedback), and thus might divide attention to a lesser degree. As such, observation training with explanation feedback should place less strain on working memory than other category-learning approaches and should therefore lead to better learning.

We tested these predictions across six laboratory experiments, in which we crossed type of training (classification vs. observation) and type of feedback (correct-answer vs. explanation). Across these experiments, we assessed learning and transfer immediately after training (Experiments 1–2), after a 10-min delay (Experiments 2–4), after a two-day delay (Experiment 5), and after a one-week delay (Experiment 6).

## Category learning and ecological validity

Although a small number of studies have investigated whether classification versus observation training leads to differential category learning and transfer, these studies have produced mixed findings, as some have shown benefits of classification over observation training (Ashby et al., 2002; Carvalho & Goldstone, 2015; Jacoby et al., 2010; Markant & Gureckis, 2014; Ramscar et al., 2010; Steininger et al., 2022; Yang & Shanks, 2018), whereas others have shown the opposite pattern (Levering & Kurtz, 2015; Patterson & Kurtz, 2020) or have failed to find direct learning differences between these training methods (Lee & Ahn, 2018). Nevertheless, it is important to note that there are numerous differences among these studies, which might account for their different outcomes. For example, some of these studies used artificial categories (Ashby et al., 2002; Patterson & Kurtz, 2020), whereas others consisted of more natural, ecologically valid categories (Jacoby et al., 2010; Lee & Ahn, 2018; Steininger et al., 2022; Yang & Shanks, 2018). Moreover, the complexity of the to-be-learned categories also varied, with some studies including relatively simple categories (e.g., categories defined by simple features; Ashby et al., 2002; Levering & Kurtz, 2015) and others including more complex categories (e.g., categories defined by relationships among various concepts; Patterson & Kurtz, 2020; Steininger et al., 2022).

It is important to reiterate that various studies have incorporated natural, ecologically valid categories that are educationally relevant (e.g., birds, rocks) and have demonstrated learning through classification training (e.g., Jones & Ross, 2011; Meagher & Nosofsky, 2023; Nosofosky et al., 2017, 2019; Wahlheim et al., 2011). A small subset of these studies has directly compared training that involves classification versus observation and have shown that the former is more beneficial for learning (e.g., Jacoby et al., 2010; Steininger et al., 2022). Thus, there is some evidence that classification training can better aid the learning and transfer of educationally relevant concepts than observation training.

Nevertheless, it is critical to note that with the exception of Steininger et al. (2022), the aforementioned experiments consisted of perceptual categories. This point is important, because for such categories, the elements that determine category membership can be directly visually perceived in a given stimulus that is fairly simple (e.g., color, size, shape, texture).

This property starkly differs from most educational concepts, which are typically taught explicitly through verbal instruction and written text. The elements that define these concepts are often relatively abstract and cannot always be directly perceived in a given scenario, as these elements can be instantiated across a wide array of objects and features (Goldwater & Schalk, 2016). For example, the elements that determine whether a given study is a true experiment will not typically be directly perceptible, as there are no surface features that can definitively indicate whether a study consists of a confound. To make such a determination, learners must typically consider how the elements within a given scenario are interconnected by their shared relations and the role that each element fills within those relations (these types of concepts are often considered to be *relational categories*; see Corral & Jones, 2012, 2014, for a formal discussion of relational categories; also see Gentner & Kurtz, 2005; also see Goldwater & Schalk, 2016, for a discussion of relational categories in education). For non-perceptual categories, students must therefore seemingly develop a somewhat abstract representation of the elements that determine category membership, which extend beyond the surface features that can be directly perceived in a given stimulus.

Historically, the work on category learning has largely focused on perceptual, feature categories (Gentner, 2005; Gentner & Kurtz, 2005; Goldwater & Markmen, 2011; Goldwater & Schalk, 2016; Markman & Stilwell, 2001). However, given that most of the more meaningful educational concepts that students learn about are not of this type and are somewhat abstract (Goldwater & Schalk, 2016), it is largely an open question about whether theories of category learning hold for non-perceptual categories, particularly those that involve educationally relevant concepts. With these issues in mind, the present set of experiments incorporate non-perceptual categories about true and non-true experiments.

The questions addressed in this paper have critical implications for learning and instruction and can directly inform instructors’ pedagogy, particularly on the issue of incorporating category learning into the classroom. It is therefore important to test these questions with ecologically valid learning materials that are of comparable complexity to the types of materials that students ordinarily learn, as the learning outcomes might differ as a function of the complexity of the materials. To this end, we examine the aforementioned questions on concepts that were taken directly from a psychology undergraduate course on research methods, which are largely identical to the concepts that are covered in most research method courses in other science-based domains. An important benefit of using these materials is thus their wide applicability to science at large.

## Experiment 1

Subjects were first asked to study a series of concepts from a basic research methods course, which was followed by a learning phase. During the learning phase, subjects were presented hypothetical experiment scenarios (see Fig. 1) and were asked to try to learn how to distinguish true from non-true experiments. Subjects who received classification training were asked to classify each scenario as either a true or non-true experiment. Based on the type of feedback that classification subjects received, after each response, they were either presented correct-answer feedback or explanation feedback. Correct-answer feedback only included the correct answer (e.g., *this is a true experiment*), whereas explanation feedback included the correct answer along with a detailed explanation (as shown in Fig. 1).

For subjects who received observation training, each scenario was presented with the corresponding category label; for those who received explanation feedback, a detailed explanation of the correct answer was also presented. Thus, the same stimuli and feedback were presented to the classification and observation training conditions. These conditions only differed by whether subjects were asked to make a classification judgment before the correct answer was shown on each trial.

**Fig. 2:**
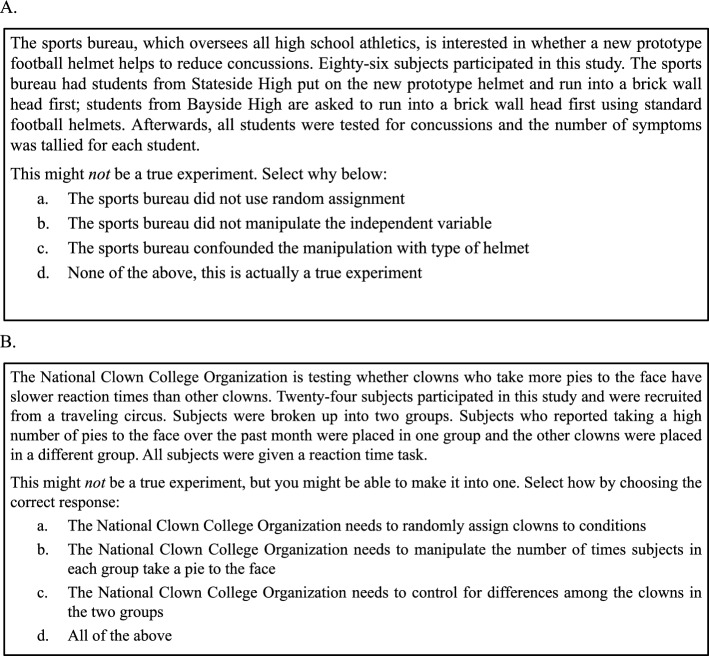
An example of a specification (Panel **A**) and reparative question (Panel **B**) used in the posttest in Experiments 1–6. Specification questions asked subjects to determine what was specifically wrong with the hypothetical scenario and reparative questions asked them to determine how to fix the scenario in order to turn it into a true experiment

After the learning phase, all subjects completed a posttest that consisted of *classification questions*, wherein subjects were asked to classify novel hypothetical experiment scenarios. These questions were followed by *application* questions, which were multiple-choice questions that involved novel hypothetical experiment scenarios and subjects were either required to identify the specific reason that a given scenario was not a true experiment (*specification questions*; see Fig. 2A) or to determine how to turn the scenario into a true experiment (*reparative question*; see Fig. 2B).

It is important to note that both of these question types allow us to assess the transfer of learning. The classification questions assess a more proximal form of transfer because this question format corresponds to the format that was presented during the learning phase. In contrast, the application questions assess a more distal form of transfer, as this question format is different from the format that was used during the learning phase and requires subjects to specify what is wrong with a given hypothetical experiment scenario or to figure out how to fix it.

## Methods

### Subjects

Two hundred sixty-nine subjects participated in this experiment for course credit in an introductory psychology course at Iowa State University (ISU). Experiments 1, 2, 5, and 6 were approved by the Institutional Review Board (IRB) of ISU, and Experiments 3 and 4 were approved by the IRB of University of Colorado Boulder.

The data collection plan for Experiments 1–6 was to run the maximum number of subjects that could be recruited in the semester that the experiment was conducted. The criterion for stopping data collection was based on an a priori power analysis with approximately 80% power to detect a medium effect size (*f* = 0.25; *alpha* = 0.05) in posttest performance among the training conditions. Thus, once the semester was over, if the sample size was large enough to meet this criterion, data collection for the corresponding experiment was stopped. For Experiments 1–6, this criterion was met during the semester that each experiment was run.

### Design

Subjects were randomly assigned to one of four training conditions: classification training/correct-answer feedback (*n* = 67), classification training/explanation feedback (*n* = 68), observation training/correct-answer feedback (*n* = 66), and observation training/explanation feedback (*n* = 68). The posttest, including novel classification and application questions, occurred immediately after training.

### Materials and procedure

#### Pretest quiz and study phase

We note that all of the materials were taken directly from Corral & Carpenter, (2020; also see Corral et al., 2021). The stimuli and instructions were presented at the center of the screen on a black background and subjects entered their responses on a computer keyboard. To assess subjects’ prior knowledge about the material, they were first asked to complete an 8-item multiple-choice pretest quiz (labeled a-e). This pretest covered general research method concepts (e.g., third variable problem, reverse causation).

Subjects were then presented five PowerPoint-style slides about basic research method concepts, which are necessary to assess whether a study meets the criteria of a true experiment; these slides were used by Corral & Carpenter, (2020) and Corral et al., (2021) and were modeled after the slides used by Corral et al., 2019 (also see Corral et al., 2022). The following concepts were covered in each of the corresponding slides: (a) Slide 1 (independent and dependent variables), (b) Slides 2 and 3 (true and non-true experiments, correlations, and causal inference issues), (c) Slide 4 (experimental control and confounds), and (d) Slide 5 (random assignment).

Subjects were instructed to carefully study these slides and were notified that they would later be tested on this material. Subjects were given seven minutes to study these slides, but the amount of time that they could spend on each slide was self-paced. Subjects could move to the next slide by pressing the ‘N’ key and to the previous slide by pressing the ‘B’ key; these instructions were displayed in a prompt directly above each slide. A slide tracker was presented at the bottom of the screen, which notified subjects which slide they were viewing (e.g., *Slide 2/5*). If subjects tried to move beyond the fifth slide prematurely (i.e., before seven minutes), the screen was cleared, and they were instructed to continue to study for the duration of the study time that remained and were asked to press the spacebar to return to the previous slide (i.e., Slide 5).

#### Learning phase

Subjects were then presented twelve hypothetical experiment scenarios (one at a time). Half of these scenarios were true experiments and half were non-true experiments. For the non-true experiments, two had a confound, two lacked random assignment, and two lacked a manipulation; see Corral & Carpenter, (2020) for a more detailed explanation on how these stimuli were constructed and sampled. The order in which the scenarios were presented was randomized for each subject.

Subjects who received classification training were presented a prompt directly above each scenario that instructed them to press ‘Y’ if the scenario was a true experiment or ‘N’ if it was not. After subjects entered a response, feedback was presented in green text directly beneath the scenario. For subjects who received correct-answer feedback,^2^ they were only shown whether the scenario was a true or non-true experiment, whereas subjects who received explanation feedback were also presented an explanation of the correct answer (see Fig. 1).

The scenario and the feedback remained on the screen for 30 s and subjects were instructed to study both carefully and that they would be notified when it was time to move on, after which a prompt was presented directly beneath the feedback, instructing subjects to press the spacebar to continue. The screen was then cleared for 300 ms, which was followed by the subsequent trial.

A similar procedure was used for subjects who received observation training, but unlike with classification training, these subjects were not asked to make a classification judgment. Instead, on each trial subjects were presented a hypothetical experiment scenario along with a prompt that instructed them to press ‘Y’ to see the correct answer (see Patterson & Kurtz, 2020 for a similar procedure for observation training). Once subjects pressed the Y key, they were shown the same feedback that was presented to subjects in the classification conditions.

#### Posttest

After completing the learning phase, subjects were immediately presented instructions notifying them that they would be tested on the material that they had just learned about. First, subjects were presented 12 novel classification questions, in which six were true experiments and six were non-true experiments; as in the learning phase, for the non-true experiment scenarios, two contained a confound, two did not incorporate random assignment, and two lacked a manipulation.

After completing this classification task, subjects were presented 12 application, multiple-choice questions (labeled a-d). All application questions consisted of scenarios that were non-true experiments. Six of these scenarios asked subjects to select the option that indicates what was specifically wrong with the scenario (*specification* questions) and another six asked them to select the option that specifies how to turn the scenario into a true experiment (*reparative* questions; Fig. 2 shows examples of each of these question types). Both specification and reparative questions consisted of two scenarios that involved a confound, two that lacked random assignment, and two that did not include a manipulation.

All subjects received the same classification and application questions on the posttest. The order in which classification and application questions were presented was randomized for each subject. After subjects entered a response, the screen was cleared and a ‘Thank You’ prompt was presented at the center of the screen, which was shown for 500 ms.

## Results and discussion

First, we note that for Experiments 1–6, performance on the pretest quiz positively predicted performance on the classification and application questions, such that subjects who performed better on the pretest quiz also performed better on the posttest (all *p*s < 0.001). However, for Experiments 1–6, no performance differences were found among any of the conditions on the pretest quiz (all *ps* > 0.103). Thus, we do not include pretest scores as a covariate in any of the reported analyses.^3^

Table 1 shows mean performance on classification and application questions, partitioned by training and feedback type in Experiments 1–6. To remind the reader, the classification questions assess a more proximal form of transfer, whereas the application questions assess a more distal form. To directly test and clearly interpret how each condition impacts these different types of transfer, we thus analyze performance on the classification and application questions separately.

**Table 1:** Each condition’s mean performance in the learning phase and on the posttest classification and application questions in Experiments 1–6

	Classification training		Observation training	
	Correct-answer	Explanation	Correct-answer	Explanation
Experiment 1				
Learning phase	0.621 (0.020)	0.629 (0.020)	–	–
Classification	0.606 (0.022)	0.703 (0.024)	0.530 (0.024)	0.705 (0.022)
Application	0.340 (0.027)	0.430 (0.028)	0.323 (0.022)	0.387 (0.025)
Experiment 2				
Learning phase	0.598 (0.202)	0.641 (0.019)	–	–
Classification	0.562 (0.020)	0.718 (0.021)	0.570 (0.019)	0.661 (0.020)
Application	0.315 (0.025)	0.364 (0.027)	0.292 (0.019)	0.403 (0.025)
Experiment 3				
Learning phase	0.641 (0.018)	0.667 (0.019)	–	–
Classification	0.594 (0.024)	0.683 (0.021)	0.622 (0.022)	0.669 (0.022)
Application	0.396 (0.028)	0.381 (0.023)	0.376 (0.025)	0.440 (0.023)
Experiment 4				
Learning phase	0.626 (0.017)	0.675 (0.019)	–	–
Classification	0.631 (0.019)	0.654 (0.019)	0.575 (0.020)	0.654 (0.021)
Application	0.403 (0.021)	0.395 (0.024)	0.379 (0.023)	0.425 (0.025)
Experiment 5				
Learning phase	0.614 (0.023)	0.667 (0.025)	–	–
Classification	0.589 (0.023)	0.654 (0.025)	0.583 (0.031)	0.625 (0.025)
Application	0.322 (0.029)	0.430 (0.039)	0.384 (0.034)	0.383 (0.038)
Experiment 6				
Learning phase	0.650 (0.023)	0.673 (0.023)	–	–
Classification	0.571 (0.030)	0.603 (0.027)	0.602 (0.033)	0.644 (0.028)
Application	0.333 (0.028)	0.362 (0.025)	0.383 (0.034)	0.371 (0.029)
Experiments 1–6				
Learning phase	0.624 (0.008)	0.657 (0.008)	–	–
Classification	0.595 (0.009)	0.675 (0.009)	0.578 (0.010)	0.663 (0.009)
Application	0.355 (0.011)	0.392 (0.011)	0.352 (0.010)	0.405 (0.011)

Standard errors of the mean are shown in parentheses

For our primary analyses, we report two separate two-way analyses of variance (ANOVA) models, with training (classification vs. observation) and feedback type (correct-answer vs. explanation) as between-subject factors. Performance on the classification questions is included as the dependent measure for one of these models and performance on the application questions is included as the dependent measure in the other model.

### Classification questions

The results reveal no main effect of training on the classification questions, *F*(1,265) = 2.570, *p* = 0.110, *MSE* = 0.036, ${\upeta }_{\text{p}}^{2}$ = 0.010. No interaction occurred between training and feedback type, *F*(1,265) = 2.742, *p* = 0.099, *MSE* = 0.036, ${\upeta }_{\text{p}}^{2}$ = 0.010. Additionally, a main effect of feedback type was found, *F*(1,265) = 34.55, *p* < 0.001, *MSE* = 0.036, ${\upeta }_{\text{p}}^{2}$ = 0.115, such that subjects who received explanation feedback performed better on the classification questions than subjects who received correct-answer feedback.

### Application questions

No main effect of training was found on the application questions, *F*(1,265) = 1.323, *p* = 0.251, *MSE* = 0.045, ${\upeta }_{\text{p}}^{2}$ = 0.005, and no interaction was observed between training and feedback type, *F*(1,265) = 0.266, *p* = 0.606, *MSE* = 0.045, ${\upeta }_{\text{p}}^{2}$ = 0.001. However, as with the classification questions, a main effect of feedback type was found, *F*(1,265) = 9.012, *p* = 0.003, *MSE* = 0.045, ${\upeta }_{\text{p}}^{2}$ = 0.033, as subjects who received explanation feedback performed better on the application questions than subjects who received correct-answer feedback.

## Summary

The findings failed to yield a statistically reliable performance difference between classification and observation training, and no interaction occurred between training and feedback type. Critically, however, as observed in previous experiments (Corral & Carpenter, 2020; Corral et al., 2021; also see Butler et al., 2013), explanation feedback led to better learning and transfer than correct-answer feedback. This finding is important, as it indicates that the null findings were not due to floor effects, wherein subjects simply failed to learn, as subjects were indeed able to learn and transfer the concepts that they were being taught. Instead, the present findings suggest that both classification and observation training produce similar levels of learning and transfer.

## Experiment 2

Although Experiment 1 failed to find any reliable performance differences between classification and observation training, it is possible that the procedure for observation training might have diminished the differences between the training conditions. Specifically, subjects who received observation training were shown each hypothetical experiment scenario and were then asked to enter a keypress to see the correct answer. It is possible that even though these subjects were not asked to make overt classification judgments, they may have nevertheless made covert judgments prior to seeing the correct response. If this is the case, the learning differences that might emerge between the training conditions would certainly be expected to be reduced, as subjects in both conditions would have essentially received classification training.

If subjects who received observation training were testing themselves covertly during the learning phase, then their response times to view the correct answer on each trial should be similar to the learning phase classification response times. Conversely, subjects who did not test themselves should have responded more quickly than subjects who did. Thus, if subjects who received observation training were not covertly testing themselves during the learning phase, their response times for viewing the correct answer on each trial should be faster than the learning phase classification response times. In line with this latter idea, subjects who received observation training (*M* = 20.17_ s_, *SE* = 1.030) in Experiment 1 had reliably faster learning phase response times for viewing the correct answer than the learning phase classification response times (*M* = 26.64_ s_, *SE* = 0.946; *p* < 0.001).

Nevertheless, to address this issue more directly, a second experiment was conducted, which used similar procedure to Experiment 1, but rather than having subjects in the observation training conditions enter a keypress to see the correct answer, the correct answer was presented simultaneously along with the corresponding scenario. This change was made to reduce the likelihood that subjects would covertly test themselves before viewing the correct answer. All other aspects of Experiment 2 were identical to Experiment 1.

## Methods

Three hundred subjects participated for course credit in introductory psychology course at Iowa State University: classification training/correct-answer feedback (*n* = 70), classification training/explanation feedback (*n* = 80), observation training/correct-answer feedback (*n* = 75), and observation training/explanation feedback (*n* = 75).

## Results and discussion

### Classification questions

As in Experiment 1, a two-way ANOVA revealed no main effect of training on the classification questions, *F*(1,296) = 1.460, *p* = 0.228, *MSE* = 0.030, ${\upeta }_{\text{p}}^{2}$ = 0.005 and no interaction between training and feedback type, *F*(1,296) = 2.597, *p* = 0.108, *MSE* = 0.030, ${\upeta }_{\text{p}}^{2}$ = 0.009. However, a main effect of feedback type was observed, *F*(1,296) = 37.831, *p* < 0.001, *MSE* = 0.030, ${\upeta }_{\text{p}}^{2}$ = 0.113, as performance on the classification questions was better for subjects who received explanation feedback than for subjects who received correct-answer feedback.

### Application questions

These same pattern of results were observed for the application questions, as no main effect of training was observed, *F*(1,296) = 0.115, *p* = 0.735, *MSE* = 0.045, ${\upeta }_{\text{p}}^{2}$ = 0.000, nor did an interaction occur between type of training and type of feedback, *F*(1,296) = 1.665, *p* = 0.198, *MSE* = 0.045, ${\upeta }_{\text{p}}^{2}$ = 0.006. Nevertheless, subjects who received explanation feedback once again performed better on the application questions than subjects who received correct-answer feedback, *F*(1,296) = 0.10.617, *p* = 0.001, *MSE* = 0.045, ${\upeta }_{\text{p}}^{2}$ = 0.035.

## Summary

To reduce the chances that subjects who received observation training were testing themselves during the learning phase by covertly making classification judgments before viewing the correct response on each trial, in the present experiment these subjects were presented the hypothetical experiment scenarios along with the corresponding feedback simultaneously. Nevertheless, our results replicated those of Experiment 1, as type of training did not seem to lead to differential learning and transfer. On the other hand, explanation feedback was once again superior to correct-answer feedback, which indicates that the learning phase was successful in helping subjects learn and transfer the to-be-learned concepts to novel scenarios, but this benefit was similar across classification and observation training.

## Experiment 3

As noted earlier, classification training incorporates principles of retrieval practice (Butler, 2010; Carpenter, 2009, 2011, 2012; Carrier & Pashler, 1992; Dunlosky et al., 2013; Roediger, 2013), as making classification judgments involves retrieving and updating hypotheses of the to-be-learned categories, as well as retrieving examples of the categories that have been encountered, which should aid learning and memory of this information. However, it is important to note that although the benefits of retrieval practice are quite robust, various studies have shown that its benefits are often most pronounced when the assessment of learning is not immediate but instead there is a delay between the learning phase and the posttest (Agarwal et al., 2017; for recent meta-analyses see Adesope et al., 2017; Rowland, 2014). One explanation for this finding is that when learning assessments occur immediately after the learning phase, there is not enough time for the knowledge that has been learned to decay, which obscures the benefits conferred by engaging in retrieval practice (Kornell et al., 2011).

This point is important, because in both Experiments 1 and 2 the posttest occurred immediately after the learning phase, which might have masked the learning benefits that might have been produced by classification training. To address this possibility, we conducted a third experiment, where we incorporated a 10-min delay between the learning phase and the posttest.

## Methods

### Subjects

Three hundred ten subjects participated for course credit in an introductory psychology course at the University of Colorado Boulder: classification training/correct-answer feedback (*n* = 78), classification training/explanation feedback (*n* = 77), observation training/correct-answer feedback (*n* = 79), and observation training/explanation feedback (*n* = 76).

### Procedure

This experiment was similar to Experiment 2, but differed in that after the learning phase, all subjects were given a 10-min filler task. After the learning phase, subjects were presented instructions that asked them to complete a short reading task that would take 10 min. This task involved reading comprehension, in which subjects were asked to read five short passages. For each passage, subjects were presented a multiple-choice question (labeled a-e) that assessed subjects’ comprehension of the passage. If subjects finished the task before the 10-min mark, they were presented a prompt that notified them to wait for the duration of the time that remained in the task. Once 10 min passed, the screen that subjects were on was cleared and subjects were notified that they would be tested on the information that they learned about earlier in the experiment.

All other aspects of the experiment were identical to Experiment 2.

## Results and discussion

### Classification questions

Similar to Experiments 1 and 2, a two-way ANOVA revealed no main effect of training, *F*(1,306) = 2.043, *p* = 0.154, *MSE* = 0.030, ${\upeta }_{\text{p}}^{2}$ = 0.007, and no interaction between training and feedback type, *F*(1,306) = 2.019, *p* = 0.156, *MSE* = 0.030, ${\upeta }_{\text{p}}^{2}$ = 0.007. However, a main effect of feedback type was found, *F*(1,306) = 6.471, *p* = 0.011, *MSE* = 0.030, ${\upeta }_{\text{p}}^{2}$ = 0.021, wherein subjects who received explanation feedback performed better on the classification questions than subjects who received correct-answer feedback.

### Application questions

However, unlike in Experiments 1 and 2, no main effects of training or feedback type were observed (although similar to the previous experiments, subjects who received explanation feedback numerically outperformed subjects who received correct-answer feedback), and no interaction occurred between training and feedback type on the application questions (all *p*s > 0.238 and all ${\upeta }_{\text{p}}^{2}$ = < 0.006).

## Summary

As in Experiments 1–2, we did not find evidence of learning differences between classification and observation training, nor did we observe an interaction between these types of training and type of feedback. Nevertheless, we did find evidence that learning and transfer occurred, as subjects who received explanation feedback performed better on classification questions than subjects who received correct-answer feedback; this same pattern was observed on the application questions, but did not lead to a statistically significant result.

## Experiment 4

To increase our confidence in the findings from Experiment 3, we conducted a fourth experiment, which was a direct replication of Experiment 3.

## Methods

Two hundred forty-six subjects participated for course credit in an introductory psychology course at the University of Colorado Boulder: classification training/correct-answer feedback (*n* = 63), classification training/explanation feedback (*n* = 63), observation training/correct-answer feedback (*n* = 60), and observation training/explanation feedback (*n* = 60).

## Results and discussion

### Classification questions

Once again, a two-way ANOVA revealed no main effect of training, *F*(1,242) = 0.117, *p* = 0.733, *MSE* = 0.030, ${\upeta }_{\text{p}}^{2}$ = 0.000, and no interaction between training and feedback type, *F*(1,242) = 0.865, *p* = 0.353, *MSE* = 0.030, ${\upeta }_{\text{p}}^{2}$ = 0.004. Additionally, a main effect of feedback was observed, *F*(1,242) = 9.310, *p* = 0.003, *MSE* = 0.030, ${\upeta }_{\text{p}}^{2}$ = 0.037, as subjects in the explanation feedback conditions performed better on the classification questions than subjects in the correct-answer feedback conditions.

### Application questions

As in Experiment 3, we did not find any main effects of training or feedback type on the application questions (although as in Experiment 3, subjects who received explanation feedback numerically outperformed subjects who received correct-answer feedback), and no interaction occurred between training and feedback type (all *p*s > 0.120 and all ${\upeta }_{\text{p}}^{2}$ = < 0.011).

## Summary

These findings replicate the results from Experiment 3 and are mostly in line with those from Experiments 1 and 2. No learning differences between classification and observation training were observed, nor was there an interaction between training and feedback type. There was evidence, however, of learning and transfer, as explanation feedback once again led to better performance on classification questions than correct-answer feedback. As in Experiment 3, this same pattern occurred on the application questions, but it was not statistically reliable.

## Experiment 5

Four experiments thus far failed to reveal any performance differences between classification and observation training. One possibility is that the 10-min delay between the learning phase and posttest that we used in Experiments 3 and 4 is not enough time for subjects’ knowledge to decay and may thus not be sensitive enough to detect the learning benefits that might occur from classification training. To address this possibility, we conducted a fifth experiment that incorporated a two-day delay between the learning phase and posttest. All other aspects of the experiment were identical to Experiment 4.

## Methods

One hundred ninety-six subjects participated for course credit in an introductory psychology course at ISU: classification training/correct-answer feedback (*n* = 49), classification training/explanation feedback (*n* = 50), observation training/correct-answer feedback (*n* = 49), and observation training/explanation feedback (*n* = 48).

This experiment occurred in two parts: the learning phase occurred in the first part and the posttest occurred in the second part (two days later). After the learning phase was completed, subjects were thanked for their participation and were reminded to return in two days for the second part of the experiment, in which they would be tested on the information they had just learned about.

## Results and discussion

Of the 196 subjects who participated in the first part of the experiment (i.e., the learning phase), 174 returned for the second part (an attrition rate of approximately 11%), which left the following condition samples: classification training/correct-answer feedback (*n* = 45), classification training/explanation feedback (*n* = 45), observation training/correct-answer feedback (*n* = 44), and observation training/explanation feedback (*n* = 40). All analyses were thus based on these subjects.

### Classification questions

A two-way ANOVA once more revealed no main effect of training, *F*(1,170) = 0.425, *p* = 0.515, *MSE* = 0.030, ${\upeta }_{\text{p}}^{2}$ = 0.002, and no interaction between training and feedback type, *F*(1,170) = 0.194, *p* = 0.660, *MSE* = 0.055, ${\upeta }_{\text{p}}^{2}$ = 0.001. Furthermore, a main effect of feedback type was found, *F*(1,170) = 4.110, *p* = 0.044, *MSE* = 0.030, ${\upeta }_{\text{p}}^{2}$ = 0.024, wherein explanation feedback led to better performance on the classification questions than correct-answer feedback.

### Application questions

No main effects of training or feedback type were found on the application questions, and no interaction was observed between training and feedback type (all *p*s > 0.127 and all ${\upeta }_{\text{p}}^{2}$ = < 0.015).

## Summary

As in Experiments 1–4, Experiment 5 found no reliable evidence that classification training leads to better learning and transfer than observation training, nor did we find evidence that this outcome varies as a function of the type of feedback that subjects receive. We do, however, continue to find evidence that learning and transfer are occurring, as subjects who received explanation feedback performed better on the classification questions than subjects who received correct-answer feedback; this same difference was also numerically found on the application questions, but it was not statistically reliable.

## Experiment 6

Contrary to the predictions that seem to follow from the literatures on learning through hypothesis testing (Hunt, 1965; Johnson & Krems, 2001; Klahr & Dunbar, 1988; Markant, 2019), the generation effect (Chechile & Soraci, 1999), retrieval practice (Butler, 2010; Carpenter, 2009, 2011, 2012; Carrier & Pashler, 1992; Dunlosky et al., 2013; Roediger, 2013), and cognitive load theory (Sweller, 1988), the findings for Experiments 1–5 suggest that classification and observation training produce similar levels of learning and transfer. In a final attempt to test the possibility that the delay between the learning phase and posttest was not long enough to allow the information that was acquired during the learning phase to decay, and thereby reveal the learning benefits that might emerge from classification training, a sixth experiment was conducted with a longer delay. Experiment 6 was identical to Experiment 5 but incorporated a one-week delay between the learning phase and posttest.

## Methods

One hundred ninety-four subjects participated for course credit in an introductory psychology course at ISU: classification training/correct-answer feedback (*n* = 47), classification training/explanation feedback (*n* = 50), observation training/correct-answer feedback (*n* = 48), and observation training/explanation feedback (*n* = 49).

This experiment was identical to Experiment 5, but after the learning phase, subjects were reminded to return in seven days (instead of two).

## Results and discussion

Out of the 194 subjects who participated in the first part of the experiment, 180 returned for the second part (an attrition rate of approximately 7%), which led to the following condition samples: classification training/correct-answer feedback (*n* = 42), classification training/explanation feedback (*n* = 47), observation training/correct-answer feedback (*n* = 44), and observation training/explanation feedback (*n* = 47). All analyses were based on these subjects.

Unlike in Experiments 1–5, the results revealed no main effects for training or feedback type, and no interactions between training and feedback type on either classification or application questions (all *p*s > 0.219 and all ${\upeta }_{\text{p}}^{2}$ = < 0.001).

## Summary

These results are consistent with the findings from Experiments 1–5, in that classification and observation training did not produce differential benefits on learning and transfer. However, unlike in those experiments, we did not find evidence that learning occurred, as explanation feedback did not produce better performance than correct-answer feedback on either classification or application questions (although subjects who received explanation feedback did numerically outperform subjects who received correct-answer feedback on both question types).

## Combined analyses: experiments 1–6

To get a better sense of whether the present findings provide support for the idea that classification and observation training produce similar levels of learning and transfer, we report two additional sets of combined analyses. First, so as to better guard against the possibility that the present null findings are the result of these studies being underpowered, we combine our data for Experiments 1–6 and re-run our analyses. Second, to test for evidence of the null hypothesis, we report a supplementary set of Bayesian analyses on this combined dataset.

### Classification questions

A two-way ANOVA found no main effect of training on classification questions, *F*(11,475) = 2.308, *p* = 0.129, *MSE* = 0.033, ${\upeta }_{\text{p}}^{2}$ = 0.002, and no interaction occurred between training and feedback type, *F*(11,475) = 0.073, *p* = 0.787, *MSE* = 0.033, ${\upeta }_{\text{p}}^{2}$ = 0.000. However, a main effect of feedback type was observed for classification questions, *F*(11,475) = 76.462, *p* < 0.001, *MSE* = 0.033, ${\upeta }_{\text{p}}^{2}$ = 0.049, such that explanation feedback led to better performance on the classification questions than correct-answer feedback.

### Application questions

This same pattern of results was observed for application questions, as no main effect for training occurred, *F*(11,475) = 0.168, *p* = 0.682, *MSE* = 0.044, ${\upeta }_{\text{p}}^{2}$ = 0.000, and no interaction between training and feedback type occurred, *F*(11,475) = 0.516, *p* = 0.473, *MSE* = 0.044, ${\upeta }_{\text{p}}^{2}$ = 0.000. Furthermore, a main effect of feedback type was observed, *F*(11,475) = 76.462, *p* < 0.001, *MSE* = 0.033, ${\upeta }_{\text{p}}^{2}$ = 0.049, in which subjects who received explanation feedback performed better on the application questions than subjects who received correct-answer feedback.

### Bayesian analysis

The following Bayesian analyses were run with the JASP software and were set to the software’s default parameters (JASP team, 2019; van Doorn et al., 2021). To directly test for evidence of the null hypothesis, we first conducted two independent samples, Bayesian *t*-tests with training type as the independent variable; one of these tests included performance on the classification questions as the dependent measure and the other included performance on the application questions as the dependent measure.

For classification questions, the results reveal moderate evidence for the null hypothesis (*BF* = 0.20), as the results were approximately 5 times more likely to occur under the null model. For application questions, we find strong evidence for the null hypothesis (*BF* = 0.062), as the results were approximately 16 times more likely to occur under the null model.

Additionally, to test for evidence of the null hypothesis for the interaction between training and feedback type, we conducted two Bayesian ANOVAs (one each for classification questions and for application questions), wherein we entered in training and feedback into the null model (so as to isolate and directly test the interaction term). For classification questions, we found strong evidence for the null hypothesis (*BF* = 0.078), as the interaction results were approximately 12 times more likely to occur under the null model. Similarly, we found strong evidence for the null hypothesis for application questions (*BF* = 0.097), as the results for the interaction were approximately 10 times more likely to occur under the null model.

## General discussion

The present paper examines whether classification versus observation training leads to differences in learning and transfer, and whether this outcome varies as a function of subjects receiving feedback that involves only the correct answer versus feedback that also includes an explanation. Across six laboratory experiments (also see combined analysis), we find that no such learning differences emerged, as both types of training produced similar levels of learning and transfer. These results were observed with an immediate posttest (Experiments 1–2), as well as a posttest that occurred 10 min (Experiments 3–4), two days (Experiment 5), and one week (Experiment 6) after the learning phase.

Critically, these null findings appear to be the result of both training conditions conveying similar learning benefits (and not simply subjects failing to learn). We point to two pieces of evidence that support this conclusion. First, the most direct evidence for this takeaway comes from the consistent main effect that was observed for feedback type (Experiments 1–5 and combined analysis), as subjects who received explanation feedback better learned and transferred the concepts from the materials than subjects who received correct-answer feedback. Stated more directly, when explanation feedback was used, subjects who received classification or observation training outperformed subjects who received correct-answer feedback and trained with classification or observation. These findings definitively demonstrate that subjects were indeed able to learn and transfer the to-be-learned concepts from the experiments, ruling out floor effects or manipulation failures as potential explanations for the lack of effect of training conditions.

Second, the procedures in the classification-explanation feedback conditions are identical to a set of conditions (referred to as the *mixed-explanatory feedback conditions*) from Corral and Carpenter, (2020), which involved classifying correct and incorrect examples of true and non-true experiments; these studies also used the same materials as in the present paper. Corral and Carpenter showed that subjects in these conditions consistently outperformed control subjects (who only studied PowerPoint style slides of the to-be-learned materials, but were not presented hypothetical experiment scenarios to classify or study) on classification and application questions. Thus, previous work has established that the procedures in the classification-explanation feedback conditions used in the present paper do indeed facilitate learning and transfer with the materials used in the present set of experiments. For these reasons, coupled with the Bayesian analyses we report in the Combined Analyses section of the paper, which show direct support for the null hypothesis, it directly follows that classification and observation training produced comparable learning and transfer across all six of our experiments.

As noted earlier in the paper, although there is a dearth of research that has examined how classification and observation training impact learning and transfer, the few studies that have been conducted have largely produced inconsistent findings. Specifically, some of these studies have found some support for the benefits of classification over observation (Ashby et al., 2002; Carvalho & Goldstone, 2015; Estes, 1994; Jacoby et al., 2010; Love, 2002; Markant & Gureckis, 2014; Ramscar et al., 2010; Steininger et al., 2022; Yang & Shanks, 2018), others have shown benefits of observation over classification (Levering & Kurtz, 2015; Patterson & Kurtz, 2020), and at least one other paper has found no direct learning differences between these training methods (Lee & Ahn, 2018).

It is important to note, however, that many of the aforementioned studies have used far more complex training procedures than those used here (e.g., using co-presented examples on each trial; see Patterson & Kurtz, 2020). Furthermore, the stimuli that were used in these studies varied considerably, with some involving categories defined by features (Ashby et al., 2002; Levering & Kurtz, 2015) and others involving categories defined by relations among objects and concepts in a stimulus (e.g., Patterson & Kurtz, 2020). The complexity and ecological validity of these materials also varied considerably across these experiments, with most consisting of artificial categories (e.g., Ashby et al., 2002; Levering & Kurtz, 2015; Patterson & Kurtz, 2020) and others including more real-world concepts (e.g., painting styles from different artists; Lee & Ahn, 2018; Yang & Shanks, 2018).

In the present paper, we used a more direct approach to examining the differences between classification and observation training, so as to directly isolate the effects of each training method. We also used more complex, ecologically valid learning materials than many of these previous studies, as we were primarily interested in examining how the two training procedures impact the learning of educational concepts. Using consistent materials under carefully controlled conditions, the current study contributes important new data showing that classification training did not benefit learning or transfer more than observation training, and did not interact with feedback type. Furthermore, these results occurred whether the posttest came immediately after training, or was delayed by as long as one week after training.

## Expertise reversal effect

We do note that of the few extant studies that have examined how classification and observation training impact learning and transfer, the stimuli used by Steininger et al., (2022) are perhaps the closest in complexity and ecological validity to those used in the current experiments, as they used a multitude of STEM-based concepts from various domains. Although Steininger et al. found a benefit of classification over observation training, we note an important difference between the subjects in their experiment and those reported here. The subjects from Steininger et al.’s experiment were student-teachers who all had a bachelor’s degree and were enrolled in a master’s training program for teachers, whereas the subjects in our experiments were mostly freshman undergraduate students.

This difference is important, because given their level of academic experience and achievement, the subjects from Steininger et al., (2022) might have had intermediate level knowledge on the material that they were learning about. In contrast, due to the low-level of academic experience for the subjects in our experiments, they were more likely to have little familiarity with the content that they were learning about and would likely be considered to be novices on the to-be-learned topics.

The discrepant findings reported here and those reported by Steininger et al., (2022) might thus point to an expertise reversal effect, wherein the training methods that are most effective for learning varies as a function of the learners’ level of expertise, as more expert learners often gain greater benefits from training procedures that can otherwise place a high strain on working memory for less experienced learners (Kalyuga, 2007; Kalyuga et al., 2003; Renkl & Atkinson, 2003). As a result, the procedures that are effective for more experienced learners are often less effective or even counterproductive for novices (e.g., learning through problem-solving practice; Cooper & Sweller, 1987; Sweller & Cooper, 1985; see also Carpenter et al., 2016, 2020). Hence, it is possible that for more novice learners, classification and observation produce similar levels of learning and transfer, but that classification is more effective for more experienced learners.

Nevertheless, despite subjects’ lack of familiarity with the materials, we do note that in Experiments 1–6, the learning phase performance for subjects who received classification training ranged from approximately 60–68% (see Table 1), which was well above chance (for all experiments, all *p*s < 0.001). These findings suggest that subjects made a reasonable effort to learn the study materials and that they were in fact able to do so reasonably well. This latter takeaway is particularly noteworthy given the complexity of the materials, as being able to determine whether a given study is a true experiment is a primary goal in courses on research methods, and many motivated students are still not able to do so after an entire semester of instruction.

In sum, although the present results demonstrate that both training methods produce similar levels of learning and transfer, it is likely that this effect varies as a function of various other training strategies that are incorporated into classification and observation training (e.g., one- vs. two-item classification, between- vs. within-category comparison). This effect might also depend on the properties of the stimuli that are being learned (e.g., featural vs. relational categories), as well as learners’ prior knowledge about the to-be-learned content (e.g., novices vs. intermediates vs. experts). Although addressing these questions is beyond the scope of the present paper, it will be important for future research to address how such factors might interact with the learning benefits that are produced by classification and observation training.

## Theoretical implications

In both classification and observation training, learners were presented with identical information. It therefore seems feasible that both types of training would produce similar learning benefits. However, these training methods seem to utilize different learning strategies, which based on various learning theories and principles, should lead to differential learning and transfer.

To remind the reader, classification seems to better utilize the benefits of *hypothesis testing* (Hunt, 1965; Johnson & Krems, 2001; Klahr & Dunbar, 1988; Markant, 2019), generation (Chechile & Soraci, 1999), *retrieval practice* (Butler, 2010; Carpenter, 2009, 2011, 2012; Carrier & Pashler, 1992; Dunlosky et al., 2013; Roediger, 2013), and *active learning* (Freeman et al., 2014). Engaging in these activities is thought to improve engagement, attention, motivation, and memory of the to-be-learned content, which leads to the prediction that classification training should produce superior learning and transfer than observation training. On the other hand (as explained earlier), classification training might divide attention and thus may be more cognitively demanding than learning through observation training. Based on cognitive load theory (Sweller, 1988), observation training might therefore be expected to produce better learning and transfer than classification training. Nevertheless, despite a concerted and extensive effort to design experiments that would be capable of detecting learning differences between the training conditions, neither of these predictions were observed in the current data.

Although there is ample support for the efficacy of these learning principles and strategies, it is important to note that much of this research has often only included assessments of *memory-based learning*, wherein test questions can be answered through rote memorization of the learning materials (e.g., word pairs, trivia facts, definitions). In contrast, the present set of experiments primarily assessed the transfer of learning, wherein subjects were required to transfer the concepts from the learning phase to novel scenarios. Although subjects in the present experiments could certainly memorize the correct answers from the learning phase, this information would likely be distracting and insufficient for correctly answering our posttest questions. For instance, a subject might encounter the scenario in Fig. 1A in the learning phase and remember that it was a true experiment in the posttest. However, this knowledge would be of little use in determining whether the scenario in Fig. 1C is a true experiment, as the subject must apply and reason about the knowledge that they acquired in the learning phase to the specifications of the scenario at hand.

As noted in previous work, although memory is certainly necessary for the transfer of learning, on its own, it is insufficient (Butler et al., 2017). Theorists have posited that the critical component to the transfer of learning is recognizing that a given concept is applicable to the scenario at hand (Corral & Kurtz, 2024). Thus, for any two training methods to produce differential benefits in the transfer of learning, it seems necessary for one method to better help learners recognize that a to-be-learned concept is applicable across novel scenarios (Corral et al., 2023). The present findings therefore call into question whether the learning principles and strategies that are leveraged by classification and observation training provide this type of differential learning benefit.

These data add to a growing body of research, in which predictions that follow from principles of retrieval practice (which have robust support in studies that assess memory-based learning) do not directly hold with more complex tasks that involve more meaningful forms of knowledge transfer than are typically assessed in memory-based learning (e.g., Corral et al., 2023; Peterson & Wissman, 2018; Van Gog & Kester, 2012; Yeo & Fazio, 2019). Indeed, although established theories explain how engaging in retrieval can improve memory of the to-be-learned materials (see Carpenter et al., 2022), they often do not directly account for how such tasks might benefit the transfer of learning (Corral et al., 2021, 2023; Pan & Rickard, 2018). Our findings thus highlight the need for theorists to more carefully consider the extent to which a given learning theory or strategy might apply to the transfer of learning.

### Theories on category learning

Theories on category learning also lead to various predictions about how classification and observation training should impact learning and transfer. Specifically, classification training is believed to focus learners’ attention on the diagnostic elements that define the to-be-learned categories (Markman & Ross, 2003; Yamuchi & Markman, 1998, 2000). In contrast, observation training is posited to lead to a broader focus of attention than classification training, in which learners attend to a wider range of elements in the stimuli (Levering & Kurtz, 2015). Furthermore, learners who receive observation training are posited to continue to learn about the elements in the stimuli throughout training, whereas those who engage in classification training might stop learning once they discover the diagnostic elements of the to-be-learned categories, and thus ignore non-diagnostic elements (Levering & Kurtz, 2015).

When these ideas are applied to the paradigm used in the present paper, one prediction that might follow is that classification training leads to better performance on classification questions than observation training; this prediction also seems to follow from the literature on transfer appropriate processing (Morris et al., 1977), as subjects who received classification training practiced engaging in classification, which was the same task that was used to administer the classification questions on the posttest. In contrast, given that observation training is thought to lead to a broader focus of attention than classification training, it is possible that the former might help subjects better learn the correlations among the category elements than the latter, which should better aid inference-based learning (Markman & Ross, 2003). We also note that the application questions required subjects to make inferences about why the study was not a true experiment or about how to turn the study into a true experiment. Thus, one prediction that might follow is that observation training should lead to better performance on the application questions than classification training.

Nevertheless, none of these predictions materialized, and it is important to consider why. One core issue is that the large majority of the literature on category learning has used artificial stimuli that were carefully crafted to test specific theories. Moreover, the research that has shown support for these theories has largely consisted of feature-based categories (for a review of this issue, see Goldwater & Schalk, 2016; also see Gentner & Kurtz, 2005; Gentner, 2005; Goldwater & Markmen, 2011; Goldwater et al., 2011; Markman & Stilwell, 2001). Both of these elements are problematic when attempting to apply these theories to real-world, educational concepts.

First, we note that educational concepts are generally far richer and are more abstract than the types of stimuli that are typically used in category-learning experiments. Educational concepts and materials may thus not readily cohere to the types of regularities that are necessary for testing theories of category learning. As such, it is not at all clear that these theories hold when scaled up to educational concepts.

Moreover, although some extant studies on category learning have included educational concepts (e.g., Jones & Ross, 2011; Meagher & Nosofsky, 2023; Nosofosky et al., 2017; 2019; Wahlheim et al., 2011), these have been feature-based, perceptual categories. This issue is problematic because many, if not most, of the concepts that students learn about are generally abstract, relational categories (Goldwater & Schalk, 2016). Indeed, these are the types of concepts that were used in the present set of experiments.

Although theoretical work has noted important differences between relational and featural categories (Gentner & Kurtz, 2005; Markman & Stilwell, 2001), theories on category learning have largely not been extended to or tested with complex, relational categories and it is unclear if and how such theories might apply to relational categories. Thus, if category learning is to meaningfully inform learning and instruction, an important future step will be to test theories of category learning with abstract relational concepts that are directly from education.

#### Challenges for future research

One issue for such work to consider is that typical category-learning paradigms can involve many hundreds or thousands of trials (e.g., Corral & Jones, 2012, 2014; Corral et al., 2018; Goldstone, 1994; Jones et al., 2006). This approach is not generally problematic because subjects are typically asked to learn about perceptual categories, in which an entire stimulus can be processed almost immediately by looking at the screen. However, most education concepts are not of this type, as they are much more complex and must often be conveyed through written text (as in the present set of experiments). Due to concerns over exhaustion and practical time constraints, it might not be feasible for subjects to read scenarios that instantiate the to-be-learned concepts over many hundreds or thousands of trials. This issue is further exacerbated if explanation feedback is used on each trial.

For logistical purposes, future category-learning studies that involve abstract relational concepts might thus be required to significantly reduce the number of trials that subjects are presented. To ensure that learning occurs, it might be necessary for such studies to incorporate more involved learning procedures than what typical category-learning studies incorporate. For example, in the present set of studies (also see Corral & Carpenter, 2020; Corral et al., 2021), although subjects only completed 12 trials during the learning phase, they were given a short tutorial on the to-be-learned concepts beforehand, and some subjects were presented explanation feedback during the learning phase of the experiment. Several other studies have also incorporated more involved learning procedures (e.g., comparing two examples from the same category, writing out their similarities and differences, and mapping their corresponding elements) over a relatively small number of trials during category learning to facilitate learning of complex education concepts (e.g., Corral & Goldwater, 2024; Corral et al., 2020; Higgins & Ross, 2011; Quilici & Mayer, 2002; Smith & Gentner, 2014). Taken together, these studies demonstrate that it is possible to use category learning to facilitate learning of complex educational concepts over a small number of trials, so long as category learning is paired with other learning procedures.

## Short-lived benefits of explanation feedback

Lastly, we note that although the benefit of explanation feedback over correct-answer feedback was generally quite robust, this effect seemed to decrease as a function of the length of the interval between the learning phase and the posttest. Specifically, the benefits of explanation feedback were strongest when the posttest was immediate (Experiments 1–2) and weakest when the posttest occurred one week after the learning phase (Experiment 6).

These findings do not appear to be the result of issues such as a floor effect or sample sizes that were not sufficient to detect an effect. Experiments 1–5 show benefits of explanation feedback over correct-answer feedback (also see Corral & Carpenter, 2020 and Corral et al., 2021), which would not be expected to consistently arise in the presence of a floor effect. Furthermore, all of our experiments were relatively well powered, and Experiments 1–4 consisted of comparable sample sizes.

We also note that the effect sizes for type of feedback were nearly identical for both classification (both ${\upeta }_{\text{p}}^{2}\hspace{0.17em}$> 0.112) and application questions (both ${\upeta }_{\text{p}}^{2}\hspace{0.17em}$> 0.020) across Experiments 1 and 2, in which the posttest was immediate. The effect size for type of feedback notably decreases, however, in Experiments 3–6. Experiments 3–4 include a posttest that occurred after a 10-min delay, wherein this effect became notably smaller for the classification questions (both ${\upeta }_{\text{p}}^{2}\hspace{0.17em}$< 0.038) and disappeared altogether on the application questions (both ${\upeta }_{\text{p}}^{2}\hspace{0.17em}$< 0.015). The posttest occurred after a two-day delay in Experiment 5, which produced comparable effect sizes to those of Experiments 3 and 4. The posttest occurred after a one-week delay in Experiment 6, which seemed to eliminate the effect of feedback type on both classification and application questions (both ${\upeta }_{\text{p}}^{2}\hspace{0.17em}$< .001). The present findings thus point to an important possible boundary condition for explanation feedback, as its benefits might be short lived and may wane over time.

## Conclusion

Recent work has noted the important and pervasive nature of category learning in education, particularly in STEM-based domains (e.g., physics, mathematics, psychology, biology; Goldwater & Schalk, 2016; Nosofsky & McDaniel, 2019). Furthermore, is has been posited that the transfer of learning can be significantly improved in the classroom if instructors adopt category learning as a pedagogical tool (Corral & Kurtz, 2024). These ideas are particularly noteworthy, because for many decades, a central goal in education and instruction has been to find ways to improve the transfer of learning in students (Bransford et al., 2000; Ellis, 1965; Hajian, 2019; National Research Council, 2012). Despite this focus, there have been only modest gains in achieving this goal.

Critically, various studies have demonstrated that category learning can indeed be used to help learners acquire and transfer a variety of complex concepts (Corral & Carpenter, 2020; Corral et al., 2020, 2021; Higgins & Ross, 2011; Lee & Ahn, 2018; Nosofsky et al., 2018; Quilici & Mayer, 2002; Smith & Gentner, 2014; Steininger et al., 2022; Wahlheim et al., 2011; Yang & Shanks, 2018). Nevertheless, the manner in which to best improve learning and transfer through category learning is largely an open question. The present paper thus serves as an initial step to examining this question.

One important implication that follows from the present findings is that students might learn equally well using either classification or observation training. Furthermore, if the goal is to maximize learning and transfer during category learning, explanation feedback might be a better option over correct-answer feedback (although instructors and students should be mindful that the strength of this benefit might decrease over longer intervals).

Future work will be necessary to investigate whether the present findings extend to other types of learning materials from other domains in education (e.g., mathematics, physics, biology, chemistry) and to other populations of learners (e.g., novices vs. intermediates). This work should also examine how various other components in a category-learning paradigm (e.g., number of examples presented on each trial, interleaved vs. massed presentation of category examples, number of categories that are learned within a single training session) can best be modified to optimize learning and transfer.

## Data Availability

All data and stimuli presented here are available from the corresponding author upon request.

## Footnotes

1 We do note that engaging in classification training likely involves some degree of germane cognitive load, which can benefit learning (Sweller, 1988; Sweller et al., 1998). However, these benefits might be offset by various other factors that can increase extraneous load and strain working memory (e.g., focusing on superfluous features, dividing attention among various subtasks, expending cognitive resources to test and update incorrect hypotheses).

2 Correct-answer feedback was used instead of corrective feedback so as to minimize the differences between the classification and observation training conditions, as corrective feedback would provide subjects who receive classification training explicit information about whether their response was correct, which subjects who receive observation training do not receive.

3 The pattern of results and their interpretations for all of the reported analyses are the same when pretest scores are included as a covariate in the reported analyses.
